# Refining a giant virus lineage: a novel order unifying *Mamonoviridae* and “Manesviridae,” unveiled by the discovery of furtivovirus

**DOI:** 10.1128/jvi.02031-25

**Published:** 2026-05-14

**Authors:** Jiwan Bae, Masaharu Takemura

**Affiliations:** 1 Department of Mathematics and Science Education, Graduate School of Science, Tokyo University of Science 26413, Shinjuku, Tokyo, Japan; Michigan State University, East Lansing, Michigan, USA

**Keywords:** virus isolation, phylogenomics, phylum *Nucleocytoviricota*, classification, family *Mamonoviridae*, clandestinovirus, furtivovirus, nucleus

## Abstract

The evolutionary origins and taxonomic framework of giant viruses related to the family *Mamonoviridae* and its relative group, including clandestinovirus, remain unclassified due to gaps in genome size and host range between these two groups. This study aimed to address this gap by integrating our newly isolated virus with publicly available metagenome-assembled genomes (MAGs) to construct a more robust phylogenetic framework. Here, we report the isolation and characterization of a new giant virus, furtivovirus, using the unicellular amoeba *Vermamoeba vermiformis* as a host. Furtivovirus has a genome of approximately 560 kbp and shares key features with its closest relative, clandestinovirus. Ultrastructural analysis revealed a unique host-nucleus-dependent replication strategy characterized by the breakdown of the nuclear membrane and the packaging of nascent virions directly within the nucleoplasm, distinguishing it from canonical cytoplasmic virion factories. Comprehensive phylogenetic and comparative genomic analyses of shared orthologous groups and nucleocytovirus marker proteins revealed that furtivovirus, clandestinovirus, ushikuvirus, and usurpativirus form a distinct monophyletic clade, for which we propose a new family, “Manesviridae.” Further analysis using amino acid-based similarity metrics of *Nucleocytoviricota* viral genomes, including established MAGs, demonstrated that this new family is robustly placed as a sister group to the family *Mamonoviridae*. This study elucidated the evolutionary relationships between viruses with large and small genomes that possess similar virion sizes within this lineage. Based on this cumulative evidence, we propose the establishment of a new order to unify these two families, thereby expanding their diversity and clarifying the evolutionary history of this branch within *Nucleocytoviricota*.

**IMPORTANCE:**

Giant viruses challenge our traditional understanding of viral evolution, raising the question of how a single related group can diverge to infect different hosts while evolving into vastly different genome sizes and replication strategies. The family *Mamonoviridae* and its relatives epitomize this evolutionary divergence: one group possesses massive genomes, whereas the other has genomes that are less than half their size. The discovery of furtivovirus and its unique nucleoplasm-dependent replication cycle provides a critical biological context for this genomic disparity. Through deep comparative genomic analysis, we demonstrated that these seemingly disparate lineages share a cohesive evolutionary origin that is distinct from other established orders. This finding highlights the complexity of genome evolution, demonstrating that giant viruses can expand their overall genome size to adapt to uncertain environments while reducing their core essential genes, thereby providing new insights into the evolutionary pressures that shape the diversity of the virosphere.

## INTRODUCTION

Since the first isolation of *Acanthamoeba polyphaga* mimivirus in 2003 (1, 2), numerous amoeba-infecting viruses have been discovered over the past 20 years, most of which are referred to as nucleocytoplasmic large DNA viruses (NCLDVs), currently classified under the phylum *Nucleocytoviricota* (3). Initially proposed in 2001, this group was described as comprising large eukaryotic DNA viruses, including the following four viral families: *Poxviridae*, *Asfarviridae*, *Iridoviridae*, and *Phycodnaviridae* (4). Based on current knowledge, nucleocytoviruses are double-stranded DNA viruses that infect eukaryotes, with genome sizes ranging from approximately 45 kilobases (kb) to more than 2.7 megabases, encoding fewer than 100 to over 1,000 genes, and virion sizes ranging from 80 nm to 1.5 μm (5–7). Furthermore, some amoeba-infecting viruses have been reported to exceed 2 μm in individual particle size (8). Despite this immense diversity, NCLDVs possess a set of characteristic core genes. The identification of these core genes, such as nucleocytoplasmic virus orthologous groups (NCVOGs) and giant virus orthologous groups (GVOGs), alongside the continuous integration of publicly available metagenome-assembled genomes (MAGs), is crucial for comprehensively charting their evolutionary history (9–11). As of 2025, after more than two decades of research, this group has expanded into a diverse phylum organized into 3 classes, 5 orders, 1 suborder, 15 families, 9 subfamilies, 60 genera, and 134 species. Despite these taxonomic advancements, significant gaps remain, particularly regarding the family *Mamonoviridae* and its close, yet unclassified, relatives. *Acanthamoeba castellanii* medusavirus J1, isolated from a hot spring in Japan in 2019 (12), and medusavirus stheno T3, isolated from a freshwater environment in Japan in 2021 (13), were classified into the family *Mamonoviridae* in 2023 (14). Following this classification, medusavirus euryale F10 was isolated from freshwater in South Korea (15), demonstrating the diversity of the genus *Medusavirus*. Medusaviruses exhibit unique features: their genomic replication occurs strictly within the host cell nucleus, and they encode a full set of histone genes, similar to those of eukaryotic organisms (12). Meanwhile, clandestinovirus (CLV), which infects *Vermamoeba vermiformis*, shares a close phylogenetic relationship with medusaviruses, along with the unique feature of encoding a full set of histone genes like medusaviruses, yet it remains unclassified at the family level due to profound differences in genome size and host range (14, 16). Other recent vermamoeba-infecting isolates, such as ushikuvirus (USKV) and usurpativirus (USPV), also fall into this unclassified clade (17–19). Consequently, the family *Mamonoviridae* is currently not assigned to any order in the International Committee on Taxonomy of Viruses (ICTV) classification (14), leaving the systematic position of this entire viral lineage unresolved.

To bridge this taxonomic and evolutionary gap, this study aimed to characterize a newly isolated GV and integrate its genome with established reference sequences and published MAGs to reconstruct a robust phylogenetic framework. Here, we report the isolation of a furtivovirus (FTV) and detail its unique nucleoplasm-dependent replication cycle. By leveraging comparative genomics and orthologous gene networks across isolates and environmental MAGs, we clarified the complex evolutionary relationships of these viruses. Therefore, we propose the establishment of the new family “Manesviridae” to encompass FTV and its relatives, and the creation of a novel order unifying “Manesviridae” and *Mamonoviridae*.

## RESULTS AND DISCUSSION

### Isolation and ultrastructural analysis of furtivovirus

**Fig 1:**
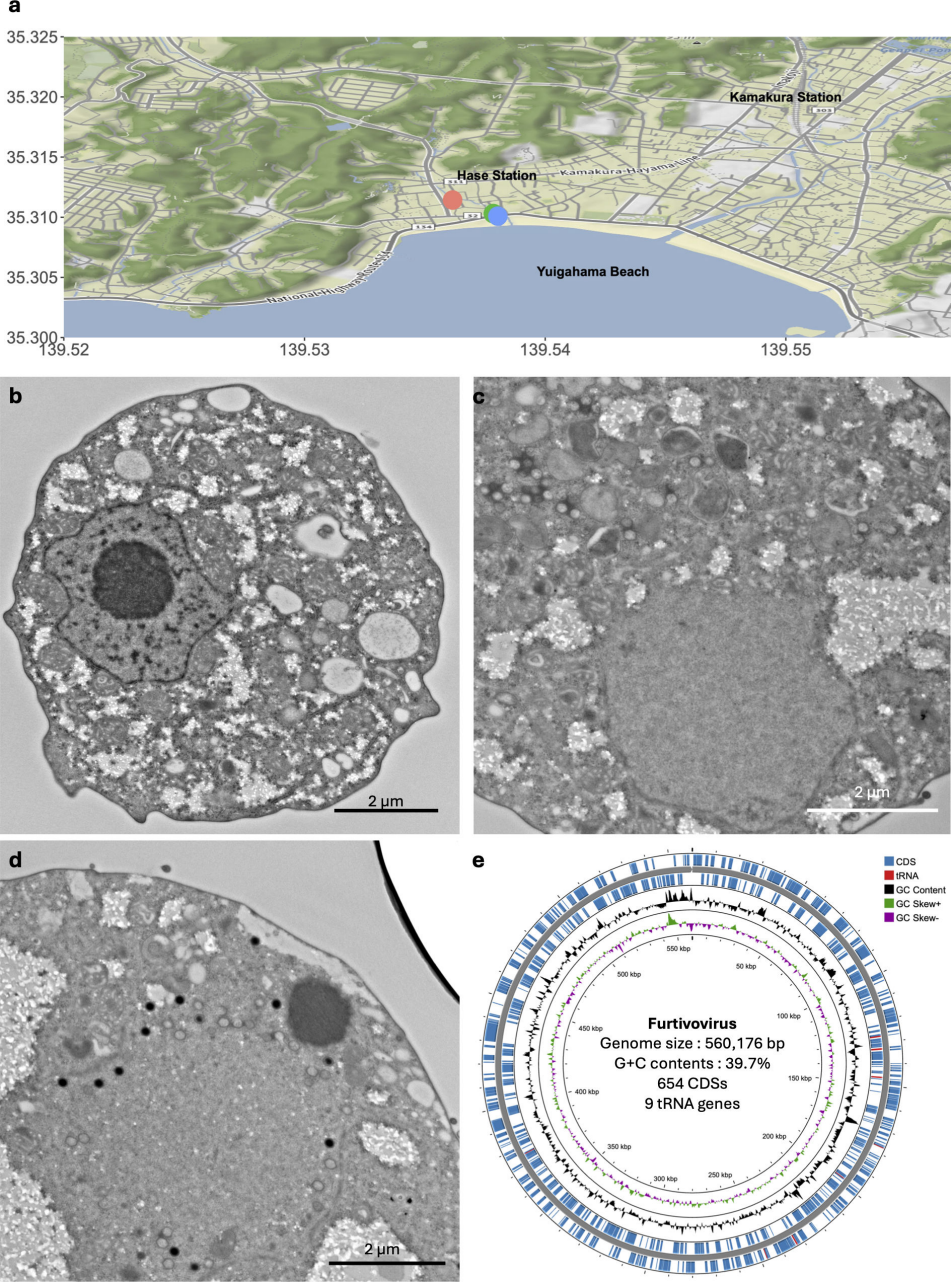
Furtivovirus isolation sites, infected cells, intracellular particle image, and its genome. (**a**) Kamakura city map showing the collection sites. The red dot represents the locations where a freshwater sample containing furtivovirus was collected. The green and blue dots represent the locations where freshwater samples were collected. A map of the collection area was generated using the ”ggmap” package in R via the Stadia Maps API. (**b**) Cell under FTV-inoculated conditions showing an intact nuclear membrane and visible heterochromatin without visible virions (possible eclipse phase). (**c**) Infected cell showing empty capsids in the cytoplasm and a vanished host nuclear membrane. (**d**) Infected cells showing capsids located in the nucleoplasm, including DNA-filled particles. Cells shown in panels b, c, and d were harvested at a multiplicity of infection (MOI) of 10 at 8 h post-infection (hpi). (**e**) The circular map represents the complete genome and features of furtivovirus.

Screening tests for GVs infecting *V. vermiformis* resulted in the observation of cytopathic effects (CPEs) following inoculation with a water sample from the Inasegawa River in Kamakura City, Kanagawa Prefecture, Japan (Fig. 1a). At this time, a conventional polymerase chain reaction (PCR) screening was performed on five wells exhibiting CPE using primers targeting the family B DNA polymerase (polB) gene of faustoviruses. This led to the identification of two faustoviruses (faustovirus sigan and faustovirus higan, unpublished) and a newly isolated virus whose particle morphology was similar to that of CLV (16). To characterize the new virus beyond its CPE, we conducted a morphological analysis. We performed conventional transmission electron microscopy (c-TEM) to observe the morphology of intracellular viral particles. The virion had a particle size of approximately 200 nm in diameter and was icosahedral, similar to CLV, USKV, and USPV (16, 17, 19).

Interestingly, our ultrastructural analysis of the infection cycle revealed distinct morphological stages that deviate from canonical giant virus replication (Fig. 1b through d). Under FTV-inoculated conditions, some host cells exhibited an intact nucleus with a clear nuclear membrane and visible heterochromatin, lacking any discernible viral particles in the cytoplasm, potentially representing the eclipse phase (Fig. 1b). However, in actively infected cells, we observed the emergence of empty capsid particles in the cytoplasm, concomitant with the breakdown and vanishing of the host nuclear membrane (Fig. 1c). While the disruption of the nuclear membrane upon virion factory formation has been previously noted in USKV (17), FTV exhibits a far more extraordinary assembly mechanism. As the infection progressed, we observed that capsids were located within the nucleoplasm (somewhat skewed toward the boundary with the cytoplasm, but entirely within the nucleoplasm without overlapping the boundary), including electron-dense, DNA-filled particles (Fig. 1d). Notably, these DNA-filled structures were not observed in the cytoplasm. This sequential observation strongly supports the hypothesis that nascent empty capsids migrate into the area of the disrupted nucleus and are packaged with viral DNA directly within the nucleoplasm. Evidence of virions migrating into the nucleus during the replication cycle of CLV has been previously reported, during which the nuclear membrane was observed to take on an abnormal appearance (16); however, this mode of genome packaging has not been explicitly observed in viruses that share the same host but assemble primarily in independent, well-defined cytoplasmic virion factories (tupanvirus, faustoviruses, and naiavirus) (8, 20, 21) or morphologically similar viruses (CLV and USKV). This finding suggests a profound host-nucleus-dependent replication and assembly strategy for FTV. Despite this unique morphology, it was difficult to fully classify the virus based solely on ultrastructural characteristics; therefore, we conducted a comprehensive whole-genome analysis.

### Whole-genome analysis and tRNA repertoire

Whole-genome analysis revealed that this new virus has a linear double-stranded DNA genome of 560,176 base pairs and contains 656 predicted coding sequences (Fig. 1e). Interestingly, FTV encodes a relatively high number of transfer RNAs (nine tRNAs) compared to its close relatives (Table 1). To understand the evolutionary rationale behind this tRNA expansion, we analyzed the anticodons encoded by FTV, CLV, USKV, and USPV in the context of the host’s (*V. vermiformis*) codon usage. According to recent host codon usage indices (22), these viral tRNAs do not correspond to extremely rare or unusually frequent host codons, nor do they appear to resolve specific codon mismatches. Instead, they correspond to codons with standard baseline usage frequencies in the host. This suggests that the retention of these tRNAs is not a strict compensatory adaptation for translation bottlenecks, but rather a clade-specific genomic signature whose precise functional benefit during the nucleoplasm-dependent replication cycle warrants further study.

**TABLE 1:** Genomic characteristics and tRNA repertoires of furtivovirus and its relatives

	Furtivovirus	Clandestinovirus	Ushikuvirus	Usurpativirus
Genome size (bp)	560,176	581,987	666,605	669,751
GC content	39.72	43.56	47.87	47.88
Number of CDSs	654	617	784	758
Encoded tRNAs (anticodon)	Leu(aag)	Ser(gct)	Val(cac)	His(gtg)
	Gln(ttg)		His(gtg)	Val(cac)
	Gln(ctg)			
	Tyr(gta)			
	Phe(gaa)			
	Gly(tcc)			
	Cys(gca)			
	Trp(cca)			
	Ser(tga)			

In addition to these genomic features, the GC content of the group of FTV, CLV, USKV, and USPV provides valuable insights when viewed through the lens of recent taxonomic discussions. The GC content of FTV, CLV, USKV, and USPV ranged from 39.72% to 47.88%. Notably, this range closely aligns with the GC content of their shared host, *V. vermiformis* CDC-19 (41.94% [GCA_045837725.1]). Conversely, members of the sister family *Mamonoviridae* (medusaviruses) exhibit a significantly higher GC content (≈60%), perfectly mirroring the GC content of their respective host, *Acanthamoeba castellanii* Neff (58.42% [GCA_046055985.1]). Such host-virus sequence composition correlations have been increasingly documented across the *Nucleocytoviricota* landscape, emphasizing that GC content often reflects specific host adaptations and biological cycles rather than strict phylogenetic constraints (23). For instance, giant viruses within the proposed family “Pandoraviridae” also exhibit a profound host-nucleus dependency during their replication cycle and possess a notably high GC content (60%–64%). As highlighted in genomic analyses of other giant viral clades (24), GC content can be highly variable and is not always a reliable, standalone taxonomic marker at the family level. Therefore, the stark divergence in GC content between *Mamonoviridae* and the group of FTV, CLV, USKV, and USPV likely reflects prolonged evolutionary adaptation to their distinct host environments. This host-driven mutational pressure further justifies their separation into two distinct families while still supporting their unification under a higher taxonomic order based on shared core replication mechanisms.

**Fig 2:**
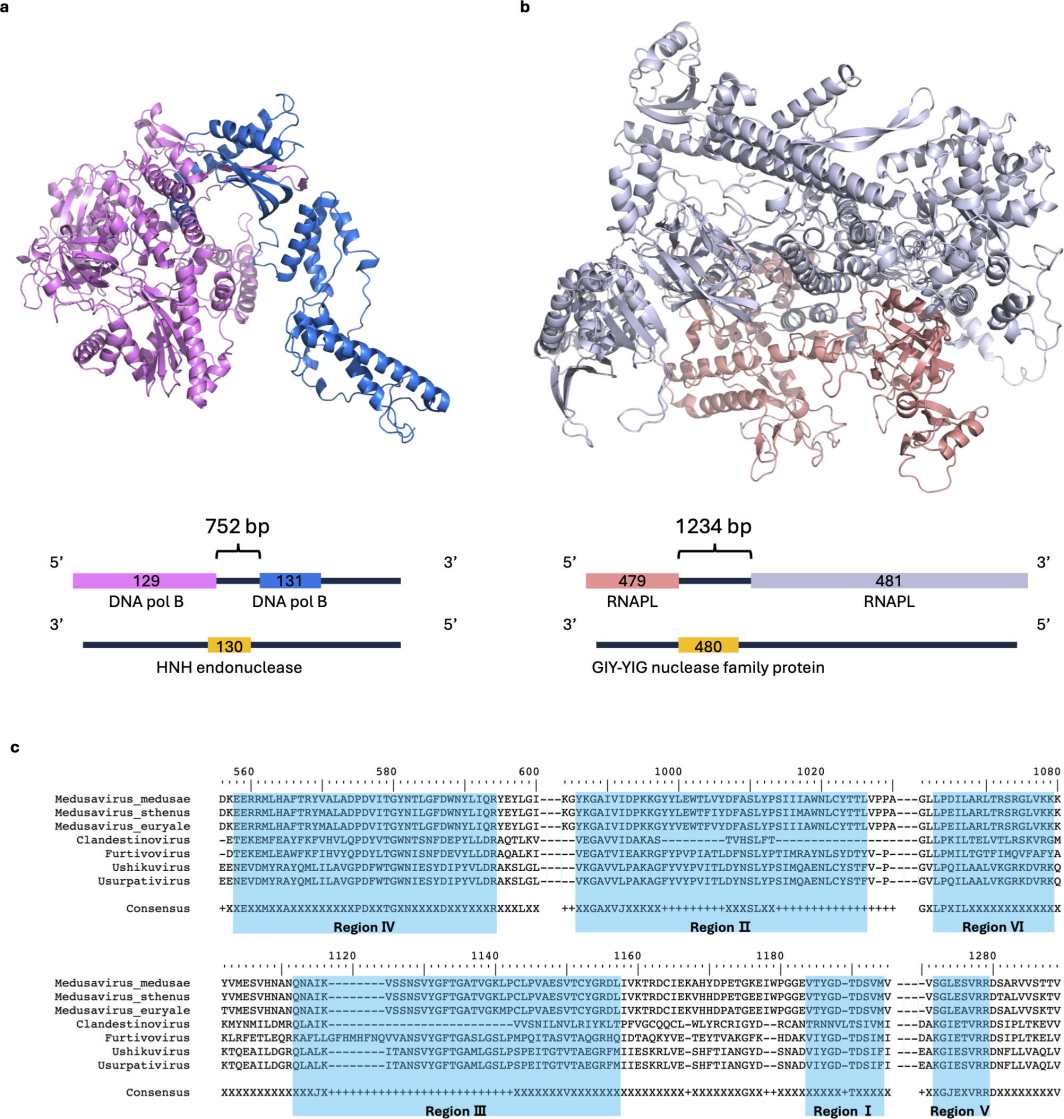
Schematic representation of protein predicted from furtivovirus amino acid sequence. Structural models of the family B DNA polymerase (**a**) and DNA-directed RNA polymerase alpha subunit (**b**) showing ORFs with introns encoding nucleases. (**c**) Amino acid sequence alignment of the expected conserved catalytic motifs of polB among the viral isolates; for the full-length alignment, refer to Fig. S2.

Shifting our focus from these broad genomic signatures to the specific genes driving these mechanisms, we find that the replication and transcription machinery of FTV exhibits notable characteristics. This new viral genome does not encode DNA topoisomerase II, like medusaviruses (12) and other relatives, but it encodes polB (open reading frames [ORF] 129, 131) and DNA-directed RNA polymerase alpha subunit (RNAPL) (ORF 479, 481), with introns similar to those of CLV (16) (Fig. 2a and b). However, unlike CLV, FTV encodes additional genes, including HNH endonuclease and GIY-YIG nuclease, on the complementary strand (Fig. 2a and b). Furthermore, unlike CLV, it encodes five HNH family homing endonucleases and three LAGLIDADG endonucleases, one of which (an HNH family homing endonuclease) showed no homology with CLV protein genes. These endonucleases may have impacted the introns present in the FTV genome (25).

### Structural analysis of the family B DNA polymerases

To evaluate the impact of these genomic differences on the core replication machinery, we modeled and compared the 3D structures of the polB using amino acid sequences and structural prediction algorithms AlphaFold3 (Fig. S1 and S2). In this process, we found significant structural differences in regions other than the protein splicing site across the family Mamonoviridae and its relatives. When the expected conserved catalytic motifs were mapped onto the primary sequences and tertiary structures (26–28), we observed that the unique secondary structural loops identified in FTV, CLV, USKV, and USPV did not intersect with the core enzymatic domains (Fig. S1 and S2). This suggests that these loops are structural additions rather than modifications of the catalytic activity. Notably, however, distinct changes in the conserved sequences were observed in CLV and FTV, with the changes being more severe in CLV. CLV lacks the YGDTDS motif in Region I and polymerase motif A (SLYPS) in Region II. In polymerase motif B in Region III, the lysine (K) residue in K...X3NS(V)YG was substituted to leucine (L), and several amino acid residues were added to the FTV gene. This motif was completely absent in CLV (Fig. 2c; Fig. S2). These severe structural mutations in the primary replicative enzyme strongly suggest that these genes are unlikely to encode functional enzymes in FTV and CLV. Coupled with the absence of topoisomerase II and the observation that viral particles migrate through the cytoplasm and enter the nucleus during CLV infection (16), this structural evidence corroborates our ultrastructural findings (Fig. 1b through d). Thus, it is highly probable that FTV and its close relatives possess impaired independent DNA replication capabilities and must rely directly on the host nucleoplasm to hijack the host’s intact replication machinery.

However, while our *in silico* structural predictions and *in vitro* ultrastructural observations provide compelling evidence for this host dependency, definitive experimental validation remains necessary. Future studies employing reverse genetics and targeted gene-editing techniques, such as knockout or precise mutation of the viral polB gene, will be crucial. Demonstrating whether FTV can successfully complete its replication cycle in the complete absence of its viral polB would explicitly determine whether this gene has degraded into a non-functional pseudogene, or whether the structurally altered protein still retains an essential, albeit non-catalytic, accessory role during nucleoplasmic genome packaging.

### Comparative genome analysis of FTV and its relatives

**Fig 3:**
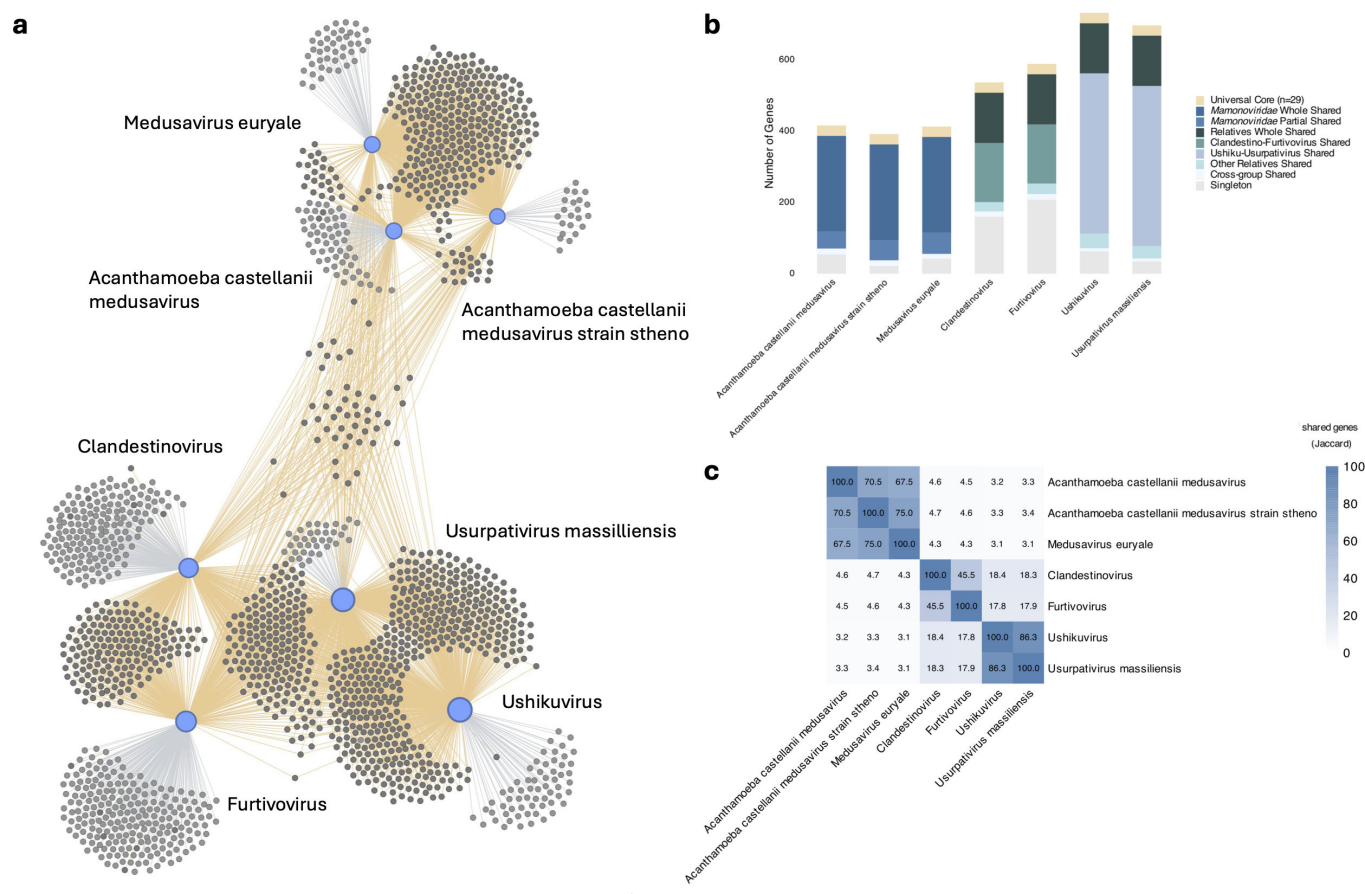
Gene sharing networks and orthologous relationships. (**a**) Bipartite network visualization of shared proteins among the seven isolates. Each viral genome is represented by a blue node, and the proteins are represented by gray nodes. Singletons are connected to the genome node by gray lines, while shared proteins are connected by wheat-colored lines. (**b**) Quantification showing the presence of only 29 universal core genes across the group. (**c**) Jaccard index matrix of shared orthogroups demonstrating distinct clustering between medusaviruses and the FTV/CLV/USKV/USPV group.

To firmly define the taxonomic boundaries among the seven isolated viruses (three medusaviruses, FTV, CLV, USKV, and USPV), we performed an extensive orthologous group (OG) analysis. First, we visualized the protein-sharing patterns using a bipartite network (Fig. 3a). This network was visibly divided into two clusters: the medusavirus group (family *Mamonoviridae*) and the CLV, FTV, USKV, and USPV group. Quantification revealed that only 29 universal core proteins were shared across all seven isolates (Fig. 3b). To contextualize this, the suborder *Ocovirineae* within the order *Pimascovirales* possesses 56 universal core genes (*Pimascovirales* universal core genes = 4), and the family *Mimiviridae* within the order *Imitervirales* shares 87 universal core genes (*Imitervirales* universal core genes = 4) in the present analysis. The extremely limited core gene set observed among our seven isolates indicates a deep evolutionary divergence that extends beyond the family level. Analysis using the Jaccard index of shared OGs clearly separated the *Mamonoviridae* group from the FTV/relatives group and confirmed the formation of two distinct sub-clusters (FTV-CLV and USKV-USPV) within the latter (Fig. 3c). To further calibrate these findings and extrapolate the taxonomic ranks of our isolates, we evaluated the Jaccard index patterns of the established order *Imitervirales* as a comparative benchmark (Fig. S3). Within *Imitervirales*, when excluding *chrysochromulina parva* virus BQ2—an extreme outlier with uniquely low gene-sharing indices across the entire order—the minimum intra-family Jaccard value observed within the family *Allomimiviridae* is 7 (Fig. S3). Furthermore, within the densely populated family *Mimiviridae*, the boundaries demarcating subfamilies typically span Jaccard values of 15–32, with the lowest internal intra-subfamily minimum reaching 11 (Fig. S3). By applying these quantitative benchmarks to seven isolates, the profound separation between the *Mamonoviridae* group and the FTV/relatives group strongly supports their classification into separate, distinct families. Subsequently, the gene-sharing indices between the two subclusters (FTV-CLV and USKV-USPV) aligned seamlessly with the established parameters for subfamily-level divergence of Mimiviridae (Fig. S3). Finally, the subtle yet persistent differences observed between FTV and CLV within their subclusters (Jaccard values of 45.5) suggest a separation of these two viruses at the genus level (Fig. 3c). Together, these metrics provide a highly resolved hierarchical framework for the taxonomy of this entire lineage.

**Fig 4:**
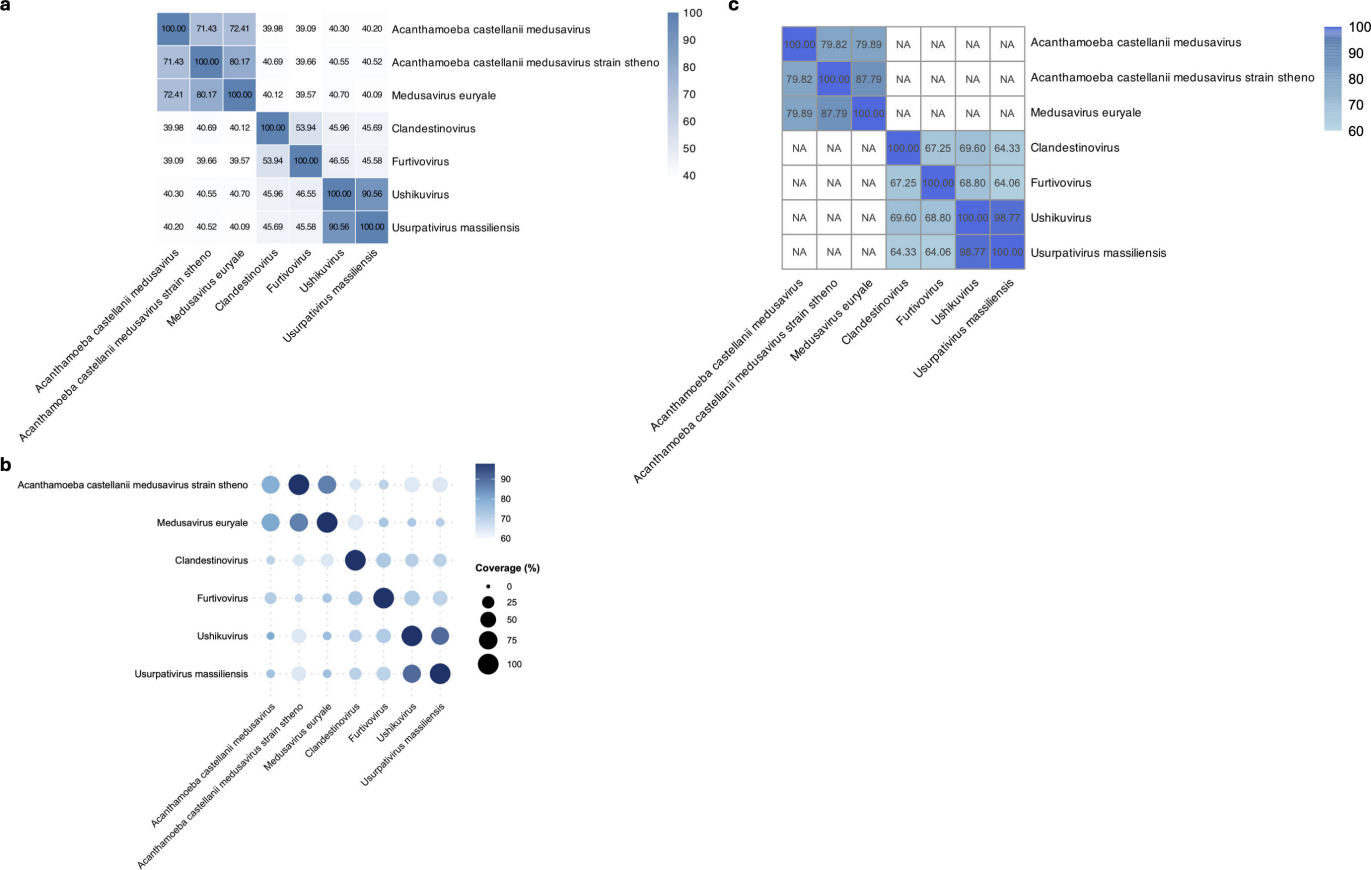
Nucleotide-level comparisons between *Mamonoviridae* and the proposed “Manesviridae.” (**a**) Gene-based average nucleotide identity (gANI) of shared orthologous genes calculated via a customized pipeline. (**b**) gANI of shared orthologs utilizing blastn. (**c**) wANI heatmap.

Because simple ortholog counts can underestimate genetic distance, we evaluated the nucleotide-level identity of the shared genes. We calculated the identity matrix of the shared OGs using a customized pipeline and blastn (Fig. 4a and b). To complement this, we performed a whole-genome average nucleotide identity (wANI) analysis, including non-coding regions (Fig. 4c). The trends of these three metrics were consistent, clearly separating the *Mamonoviridae* and FTV/relatives groups at the family level (Fig. 4). Therefore, we name the FTV/relatives group “Manesviridae.” Furthermore, these metrics showed wANI values below 70% between the clusters within the newly proposed “Manesviridae” family (Fig. 4c), supporting its subdivision into at least three distinct genera: CLV, FTV, and USKV-USPV.

### Phylogeny and the challenging of the “Pandoravirales” hypothesis

**Fig 5:**
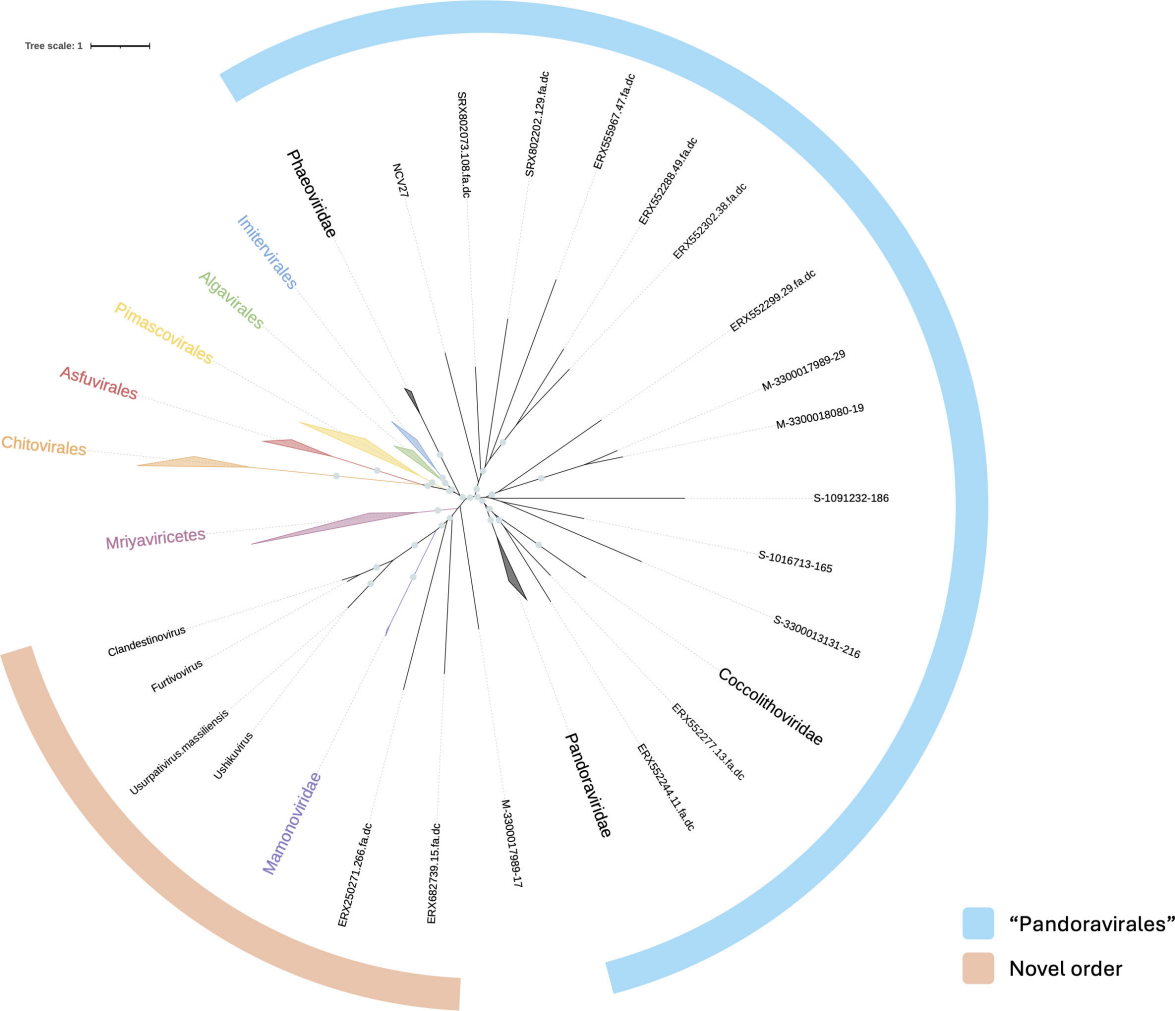
Unrooted phylogenetic tree of 372 *Nucleocytoviricota* genomes (including published MAGs) based on the concatenated amino acid sequence alignment of seven marker genes using IQ-TREE. Nodes are colored according to the ICTV-established orders/classes. Bubbles at nodes indicate ultrafast bootstrap ≥ 95% and SH-aLRT ≥ 80%.

To position FTV within the broader evolutionary landscape of the phylum *Nucleocytoviricota*, we conducted a comprehensive phylogenetic analysis using seven concatenated core marker genes extracted from 372 viral genomes (10, 29). Crucially, this data set incorporated published high-quality MAGs (29–33) to test deep evolutionary links (Fig. 5). The resulting unrooted phylogenetic tree robustly placed FTV within a monophyletic clade alongside CLV, USKV, and USPV. Furthermore, an environmental MAG ERX682739.15.fa.dc (ultrafast bootstrap ≥ 99%, SH-aLRT ≥ 89.2%) and ERX682739.15.fa.dc (ultrafast bootstrap ≥ 73%, SH-aLRT ≥ 34.3%) clustered dependably within this lineage. This entire assemblage constitutes the newly proposed family “Manesviridae,” which forms an unequivocal sister group to the family *Mamonoviridae* (Fig. 5).

In early taxonomic frameworks, it was hypothesized that medusavirus might fall under the proposed order “Pandoravirales” (10). To evaluate the validity of this hypothesis using our expanded data set, we conducted a targeted comparative analysis of universal core genes. The proposed *Mamono*-“Manes”*viridae* superclade (comprising the seven isolates and the associated MAG ERX682739.15.fa.dc) shared 11 universal core genes related to basic transcription, replication, and repair. Building upon our hierarchical Jaccard index framework, we incorporated ERX682739.15.fa.dc into this matrix confirming its distinct placement (Fig. S4). The observed gene-sharing index robustly positions this environmental MAG at the subfamily level within the *Mamono*-“Manes”*viridae* superclade, reinforcing its genetic cohesion as a discrete yet deeply related evolutionary branch. In contrast, the “Pandoravirales” group independently shares only two universal core genes (A32 packaging ATPase and polB). However, when we merged these two massive groups to test their taxonomic unity, the number of shared core genes collapsed to one (A32 packaging ATPase). Most notably, despite both groups encoding the fundamental replication enzyme polB, they are not grouped as orthologs because of severe sequence divergence. This finding suggests significant genetic discontinuity between these lineages. To further quantify this discontinuity, we assessed the nucleotide-level identity of the shared core genes between the proposed superclade and “Pandoravirales” using a gap-sensitive blastn alignment approach, identical to our intra-family evaluations. The resulting identity matrix revealed a stark separation, distinctly clustering the “Pandoravirales” members away from the proposed novel order (Fig. S5). This group-specific gene-sharing dynamic and pronounced sequence distance unequivocally indicate that merging the *Mamono*-“Manes”*viridae* clade into the order “Pandoravirales” is taxonomically inappropriate.

**Fig 6:**
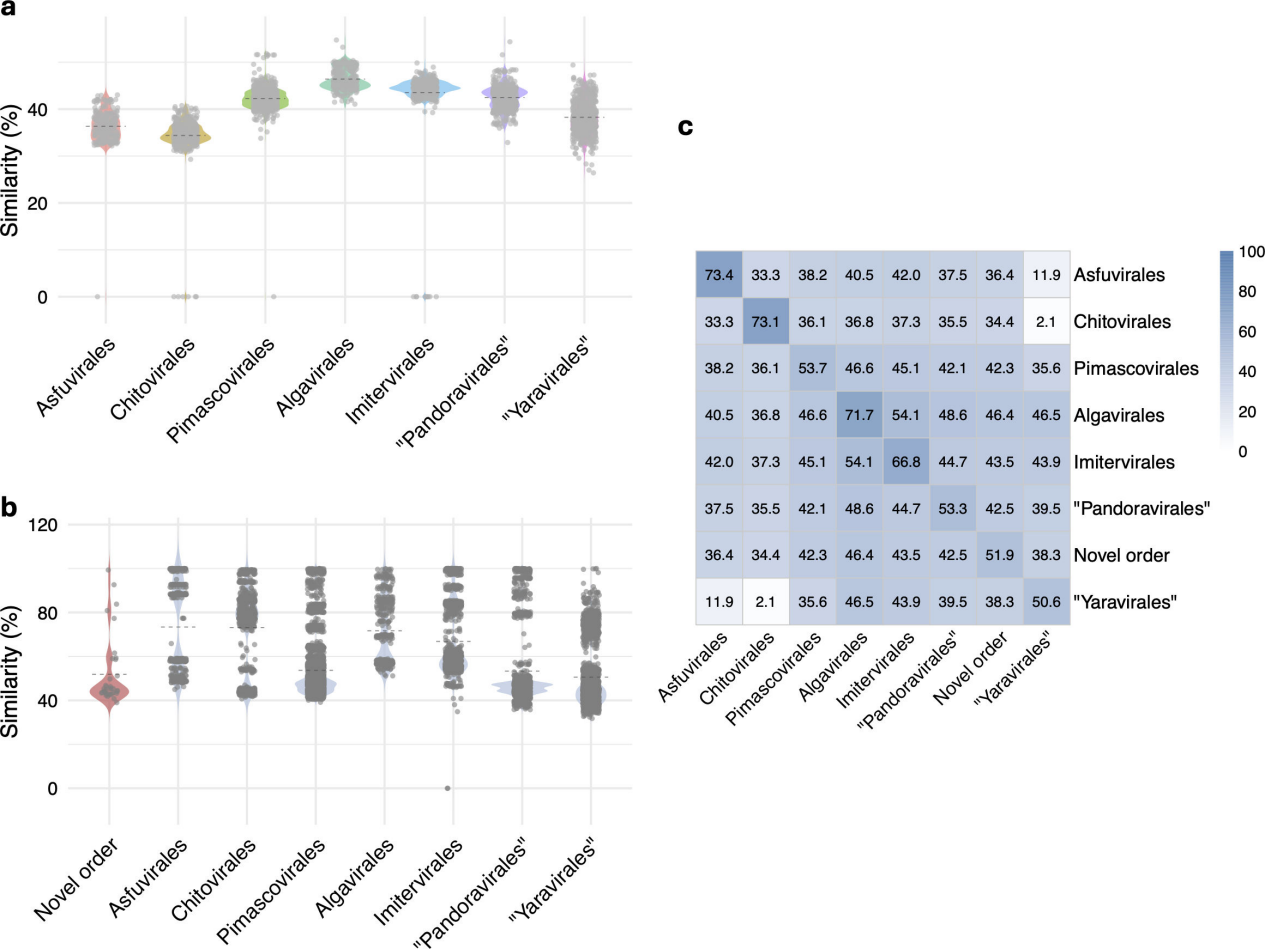
BLOSUM62-based amino acid similarity analysis of the seven marker genes across diverse viral groups. (**a**) Inter-order relationship demonstrating low similarity between the proposed order and established groups. (**b**) Intra-order diversity showing that the internal variation of the proposed order is consistent with established orders. (**c**) Heatmap summarizing the mean similarity values between taxonomic orders.

To expand our comparative framework beyond the “Pandoravirales” hypothesis and validate the evolutionary distances across the entire phylum, we assessed the amino acid similarity of the seven core marker genes across all established orders using a BLOSUM62-based identity metric (Fig. 6). This approach accounts for deep evolutionary conservation, which nucleotide-level comparisons may obscure. The inter-order comparison (Fig. 6a) definitively confirmed that the newly proposed lineage shares a consistently low baseline similarity with all other established orders (including the proposed order “Pandoravirales”). Meanwhile, the internal diversity of the nine genomes (seven isolates + two MAGs) comprising our proposed group (Fig. 6b) was consistent with the level of variation accepted within other established orders. The heatmap summarizing these relationships (Fig. 6c) provides strong quantitative evidence for establishing a distinct novel order encompassing the sister families *Mamonoviridae* and “Manesviridae.” Additionally, we analyzed relative evolutionary divergence (RED) values; however, this approach did not provide statistically significant support, limiting our ability to convincingly distinguish the proposed *Mamonoviridae* and “Manesviridae” based on evolutionary rates. This is likely because conclusively demonstrating a difference in evolutionary rates between the two families requires more genome data than is currently available to achieve sufficient statistical power.

### Conclusions

In this study, we present a comprehensive characterization of a new virus, FTV, bridging the evolutionary gap in the taxonomy of the phylum *Nucleocytoviricota*. Our ultrastructural observations clearly demonstrated a nucleoplasm-dependent genome packaging strategy for FTV, deviating from cytoplasmic virion factories and providing context for the loss-of-function structural mutations observed in its primary replication machinery. Furthermore, by integrating genomic data with existing environmental MAGs and applying multi-metric comparative genomics, we academically justified the proposal of the new family, “Manesviridae.” Crucially, the core-gene analysis results challenge the previous taxonomic hypothesis that placed this lineage within “Pandoravirales.” When these groups are merged and analyzed as a single taxon, the number of shared core genes plummets to one, and despite both groups encoding polB, they fail to group as orthologs because of sequence divergence, strongly suggesting a deep genetic discontinuity between them. Meanwhile, separating “Manesviridae” and *Mamonoviridae* into distinct families is firmly justified by the stark contrast in genome size (an approximately twofold gap), the extremely limited shared core genes (only 29), low nucleotide sequence identity comparable to distances between other established viral families, and severe structural divergence of the primary replication machinery. Furthermore, unifying these two families into a new single order is supported by their shared host-nucleus dependency and genetic cohesion within the lineage, which is comparable to other established viral orders. The stark evolutionary contrast observed within this newly proposed order—from the highly reduced, nucleus-dependent genomes of *Mamonoviridae* to the massive genomes of “Manesviridae”—provides a compelling real-world example of the dynamic, accordion-like evolution of GVs (34, 35). While some giant virus lineages expand their genomic robustness to adapt to uncertain environments (36), FTV and its relatives have adopted a strategy of extreme reductive evolution, shedding core replicative machinery to strictly parasitize the host nucleoplasm. This study not only resolves taxonomic ambiguity but also presents this clade as a useful model system for exploring the host-nucleus dependency and genome evolution mechanisms of giant viruses.

## MATERIALS AND METHODS

### Viral isolation and virion production

Fifty milliliters of freshwater samples was collected from three different locations in the Inasegawa River in Kamakura City, Kanagawa Prefecture, Japan. The 1:10 diluted water samples were inoculated and inoculated with *Vermamoeba vermiformis* CDC-19 (ATCC 50237) at 5 × 10^5^ cells per well in 96-well plates containing peptone-yeast extract-glucose medium supplemented with mixed antibiotics, as previously described (17, 37, 38). Plates were incubated at 26°C, and upon observation of CPEs, a conventional PCR screening was performed targeting the polB gene of faustoviruses and ushikuvirus. The supernatants were collected from the corresponding wells. The virions were subsequently cloned and propagated. Viral purification was performed using a 0.45 μm membrane filter, as previously described (17, 37, 38). From these isolates, we detected viral particles from two faustovirus isolates and a related clandestinovirus (CLV)-like virus, which were identified by whole-genome sequencing, as described below. We named this CLV-like isolate “furtivovirus” (FTV).

### c-TEM assay

For ultrastructural analysis, FTV-infected cells were harvested from the culture flasks at a multiplicity of infection (MOI) of 10 at 8 hpi. The harvested cells were centrifuged for 5 min at 500 × *g*. The pellet was resuspended in 1× phosphate-buffered saline (PBS) and centrifuged again for 5 min at 500 × *g*. Samples were chemically fixed five times in 2% glutaraldehyde in PBS for 30 min each and washed five times in PBS for 1 h each. The post-fixed samples were stained with 2% osmium tetroxide (NISSHIN EM Co.) for 1 h and washed twice with PBS. The fixed pellets were dehydrated through a graded ethanol series (50%, 70%, 80%, 90%, 95%, and 99.5% for 5 min each, followed by three additional treatments with 99.5% for 10 min), followed by three dehydration steps using propylene oxide for 10 min each. The dehydrated samples were infiltrated with a 1:1 mixture of EPON resin. The infiltrated sample was then exchanged twice with fresh resin, and finally, the resin sample was polymerized at 60°C for 48 h. All procedures from fixation to dehydration were performed at 4°C. c-TEM observations were performed as previously described (17, 37, 38).

### Genome analysis and tRNA annotation

Genomic DNA (gDNA) was extracted from purified FTV particles using the phenol/chloroform/isoamyl alcohol extraction method (15). The integrity of the gDNA was confirmed using agarose gel electrophoresis. The FTV genome was sequenced using the PACBIO_SMRT Revio sequencing platform, and reads were assembled *de novo* using Flye (version 2.9, https://github.com/fenderglass/Flye) by Macrogen Japan (Koto-ku, Tokyo, Japan). ORF prediction was performed using PROKKA (version 1.14.6). For functional annotation, eggNOG 4.5 and InterProScan (version 5.34-73.0) were utilized. Additionally, BLASTp searches against the NCBI non-redundant (nr) database (February 2025, e-value 10^−5^) were conducted to assess amino acid homology, followed by manual curation of the annotations. Transfer RNAs (tRNAs) were identified using ARAGORN (version 1.2.41) with the following parameters: -t (tRNA type), -i (allow intron), -l (linear topology), and -s (both strands) (39). To evaluate the evolutionary context of the identified tRNAs, the encoded viral anticodons were compared with the host (*V. vermiformis*) codon usage indices, as recently described by Willemsen et al. (22). The genome map was visualized using Proksee (40).

### Amino acid sequence and structural comparisons

The complete nucleotide (.fasta) and protein (.faa) sequences for the comparative viruses were obtained from the National Center for Biotechnology Information (NCBI). Tertiary structure predictions for polB and RNAPL proteins across the isolates were generated using AlphaFold3 (41) and visualized using PyMOL (version 3.1.3: https://pymol.org/support.html). To identify and map conserved catalytic motifs onto these structures, the primary amino acid sequences of polB from the respective viruses were aligned and visualized using the R package “DECIPHER” (42), referencing established canonical polymerase motifs (26–28).

### OG, network, and nucleotide identity analysis

OrthoFinder (version 3.1.2) (43) was used to identify OGs and calculate gene-sharing levels among viral genomes. The overall protein-sharing patterns were visualized as a bipartite network using Gephi (version 0.10.1) (44). To measure the similarity of the shared gene contents, a Jaccard index matrix was computed based on OG clustering. Because simple ortholog counts may underrepresent true genetic distances, we assessed the nucleotide-level identity of shared OGs. Two customized Python pipelines were utilized to comprehensively assess nucleotide-level similarities. First, to evaluate the identity of shared OGs, gene-based ANI coding sequences were extracted and aligned using MAFFT (45), followed by pairwise sequence identity calculations that explicitly excluded alignment gaps. To account for the presence of paralogs within a single OG, an all-vs-all pairwise comparison was conducted between the sequences of the matched genomes to calculate the average orthologous identity. A second comparison was performed using NCBI BLASTn (dc-megablast) to compute the length-weighted ANI and genomic coverage matrices. Additionally, whole-genome ANI (orthoANIu), which computes identities to include non-coding regions, was calculated (46). Matrix and heatmap visualizations were performed using R software (version 4.5.0).

### Phylogenomic reconstruction and inter-lineage similarity analysis

To reconstruct the evolutionary history of FTV, a comprehensive data set of 372 viral genomes was compiled, spanning three classes: *Megaviricetes*, *Mriyaviricetes*, and *Pokkesviricetes* (Data S1). Crucially, this data set incorporated published high-quality MAGs to bridge deep evolutionary lineages (29–33). Using the ncldv_markersearch tool (29), seven GVOGs serving as core marker genes (DEAD/SNF2-like helicase, transcription initiation factor IIB, family B DNA polymerase, RNA polymerase large subunit, packaging ATPase, DNA topoisomerase II, and poxvirus late transcription factor VLTF3) were identified and extracted. A concatenated multiple amino acid sequence alignment was generated using Clustal Omega (version 1.2.4) (47). To determine the best-fit substitution model, ModelFinder (-m TEST), built into IQ-TREE 3 (version 3.0.1) (48) was employed. The unrooted maximum-likelihood phylogenetic tree was constructed using IQ-TREE 3 with the parameters -m Q.PFAM + F + I + R9 B 1000 -alrt 1000, where -B specifies the ultrafast bootstrap (UFboot) approximation (49) and -alrt performs the SH-aLRT test (50). The resulting tree was visualized using iTOL (version 7.5.0) (51). To quantitatively validate the taxonomic distances across deeply divergent lineages independently of ortholog presence/absence, we calculated the amino acid similarity of the seven concatenated marker genes across all orders. This was performed using a custom Python 3 script employing the BLOSUM62 substitution matrix (52) to account for evolutionary conservation and gaps, providing a more robust metric than the simple sequence identity. The resulting similarity matrix was visualized as a heatmap in R. Furthermore, RED (53) values were computed from the rooted phylogenetic tree using the get_reds function from the R package “castor” (54) to assess lineage-specific evolutionary rates.

### Etymology of taxon nomenclature

FTV was named “furtivovirus” (from the Latin *furtivus*, meaning hidden or stealthy) because of its initial cryptic co-isolation alongside faustoviruses (similar to how CLV was co-isolated with faustovirus ST1 and usurpativirus with faustovirus LCD7). The proposed family name, *Manesviridae*, is derived from the *Manes*—chthonic deities or spirits of the dead in the ancient Roman religion. This nomenclature adheres to the conventions established for CLV, reflecting the observation that three of the four isolated members of this family (FTV, CLV, and USPV) were discovered as “ghostly” or secondary entities emerging in viral screenings only after the lysis caused by the faster-growing faustoviruses (18–20).

## Data Availability

The sequence data for the furtivovirus genome are available in GenBank (LC890700) under BioProject PRJDB35989 and SAMD01617881. The raw reads were obtained from the Sequence Read Archive (DRR724694).

## SUPPLEMENTAL MATERIAL

Data S1 jvi.02031-25-s0001.xlsx Virus list and raw data of BLOSUM62-based amino acid similarity analysis.

Data S2 jvi.02031-25-s0002.txt Phylogenetic tree file data text.

Supplemental figures jvi.02031-25-s0003.docx Fig. S1 to S5.

ASM does not own the copyrights to Supplemental Material that may be linked to, or accessed through, an article. The authors have granted ASM a non-exclusive, world-wide license to publish the Supplemental Material files. Please contact the corresponding author directly for reuse.
