# Supplementary material for: Refining a giant virus lineage: a novel order unifying Mamonoviridae and “Manesviridae,” unveiled by the discovery of furtivovirus
Source: J Virol. 2026 May 14;100(6):e02031-25. doi: 10.1128/jvi.02031-25 (PMC13289167; doi:10.1128/jvi.02031-25)
Supplement: Supplemental figures — Fig. S1 to S5. [file jvi.02031-25-s0003.docx]

**Supplementary Materials**

**Refining a giant virus lineage: A novel order unifying *Mamonoviridae* and “Manesviridae,” unveiled by the discovery of furtivovirus**

Jiwan Bae^a^ and Masaharu Takemura^a^*

^a^ Department of Mathematics and Science Education, Graduate School of Science, Tokyo University of Science, Kagurazaka 1-3, Shinjuku, Tokyo 162-8601, Japan

Running Head: New order of giant virus unveiled by furtivovirus

^*^ Address correspondence to Masaharu Takemura, [giantvirus@rs.tus.ac.jp](mailto:giantvirus@rs.tus.ac.jp)

**Supplemental Data 1**

Viral list and raw data of BLOSUM62-based amino acid similarity analysis

**Supplemental Data 2**

Phylogenetic tree file data text

**Supplemental Figure 1**

Comparison of polB structures from *Mamonoviridae* and “Manesviridae” families

**Supplemental Figure 2**

Comprehensive multiple-sequence alignment of family B DNA polymerases.

**Supplemental Figure 3**

Jaccard index benchmarking of shared OGs within the established order, *Imitervirales*.

**Supplemental Figure 4**

Expanded Jaccard index matrix incorporating environmental Metagenome-Assembled Genomes (MAGs).

**Supplemental Figure 5**

Nucleotide-level identity of shared core genes between the proposed novel order and “Pandoravirales.”

**Fig S1** Comparison of polB structures from the *Mamonoviridae* and “Manesviridae” families. The structure of polB from the family “Manesviridae” (shown in color; (a) clandestinovirus, (b) furtivovirus, (c) ushikuvirus, and (d) usurpativirus) was aligned with the structures of three medusaviruses: *acanthamoeba castellanii* medusavirus J1 (upper), medusavirus stheno T3 (middle), and medusavirus euryale F10 (lower). All medusavirus structural predictions are indicated in white font. α helices (red) and loops (green) unique to the family “Manesviridae” are highlighted. polB: family B DNA polymerase.

**Fig S2** Comprehensive multiple sequence alignment of family B DNA polymerases (polB). The full-length amino acid sequences of polB from the seven viral isolates were aligned using DECIPHER. Conserved catalytic regions (Regions I–VI) are highlighted. Unique secondary structural loops (Fig. S1) specific to the proposed family "Manesviridae" (furtivovirus, clandestinovirus, ushikuvirus, and usurpativirus)—which do not intersect with the core enzymatic domains—are distinctly indicated (yellow).

**Fig S3** Jaccard index benchmarking of shared OGs within the established order *Imitervirales*. To establish robust quantitative taxonomic thresholds for family and subfamily ranks, gene-sharing indices (Jaccard values) among established members of the order *Imitervirales* were determined. The matrix demonstrates typical intra-family (e.g., *Mimiviridae*, *Allomimiviridae*) and intra-subfamily Jaccard values.

** Fig S4** Expanded Jaccard index matrix incorporating environmental Metagenome-Assembled Genomes (MAGs). The gene-sharing index matrix was recalculated by integrating the robust-grouped MAG (ERX682739.15.fa.dc) associated with the *Mamono*-“Manes”*viridae* superclade.

** Fig S5** Nucleotide-level identity of shared core genes between the proposed novel order and “Pandoravirales.” A gap-sensitive BLASTn alignment approach was used to assess the sequence identity of the universally shared core genes (e.g., A32 packaging ATPase) between members of the proposed *Mamono*-“Manes” order and the “Pandoravirales” group.
